# Tranexamic Acid Promotes Murine Bone Marrow-Derived Osteoblast Proliferation and Inhibits Osteoclast Formation In Vitro

**DOI:** 10.3390/ijms22010449

**Published:** 2021-01-05

**Authors:** Anke Baranowsky, Jessika Appelt, Kristina Tseneva, Shan Jiang, Denise Jahn, Serafeim Tsitsilonis, Karl-Heinz Frosch, Johannes Keller

**Affiliations:** 1 Clinic of Trauma and Orthopedic Surgery, University Medical Center Hamburg-Eppendorf, 20251 Hamburg, Germany; a.baranowsky@uke.de (A.B.); jiangshanmed@gmail.com (S.J.); k.frosch@uke.de (K.-H.F.); 2 Julius Wolff Institute for Biomechanics and Musculoskeletal Regeneration, Charité-Universitätsmedizin Berlin, 13353 Berlin, Germany; jessika.appelt@charite.de (J.A.); kristina_tseneva@yahoo.com (K.T.); denise.jahn@charite.de (D.J.); serafeim.tsitsilonis@charite.de (S.T.); 3 Center for Musculoskeletal Surgery, Charité-Universitätsmedizin Berlin, 13353 Berlin, Germany

**Keywords:** tranexamic acid, osteoblast, osteoclast, macrophages, bone regeneration

## Abstract

Despite modern surgical trauma care, bleeding contributes to one-third of trauma-related death. A significant improvement was obtained through the introduction of tranexamic acid (TXA), which today is widely used in emergency and elective orthopedic surgery to control bleeding. However, concerns remain regarding potential adverse effects on bone turnover and regeneration. Therefore, we employed standardized cell culture systems including primary osteoblasts, osteoclasts, and macrophages to evaluate potential effects of TXA on murine bone cells. While osteoblasts derived from calvarial digestion were not affected, TXA increased cell proliferation and matrix mineralization in bone marrow-derived osteoblasts. Short-term TXA treatment (6 h) failed to alter the expression of osteoblast markers; however, long-term TXA stimulation (10 days) was associated with the increased expression of genes involved in osteoblast differentiation and extracellular matrix synthesis. Similarly, whereas short-term TXA treatment did not affect gene expression in terminally differentiated osteoclasts, long-term TXA stimulation resulted in the potent inhibition of osteoclastogenesis. Finally, in bone marrow-derived macrophages activated with LPS, simultaneous TXA treatment led to a reduced expression of inflammatory cytokines and chemokines. Collectively, our study demonstrates a differential action of TXA on bone cells including osteoanabolic, anti-resorptive, and anti-inflammatory effects in vitro which suggests novel treatment applications.

## 1. Introduction

Injury of the musculoskeletal system and major surgery often results in bleeding, which represents a high clinical challenge and requires immediate surgical attention. Although the organism in principle activates a complex system of blood coagulation cascades after vessel injury, blood loss can exceed the compensatory capacities resulting in cardiovascular shock and ultimately death [1]. Despite modern surgical techniques and invasive monitoring, excessive bleeding contributes to about one-third of trauma-related death [2]. Moreover, excessive blood loss can be observed in the case of major elective procedures including pelvic, hip, femoral, and spine surgeries.

A significant improvement was achieved through the introduction of tranexamic acid (TXA), which today is widely used in orthopedic trauma surgery to control bleeding [3]. TXA represents an anti-fibrinolytic drug that was initially developed in Japan in the 1960s [4]. Mechanistically, TXA functions as a synthetic lysine-analogue, which reversibly forms a complex with plasminogen, preventing it from binding to fibrin and therefore inhibiting the fibrinolytic pathway [2]. Its initial use was to prevent excessive menstrual bleeding; however, its potential use for other causes of bleeding, including trauma and surgery, was evaluated soon after [5]. To date, several meta-analyses elucidated the efficacy of TXA on blood transfusion requirements, and in 2010, the results of the “clinical randomization of an antifibrinolytic in significant hemorrhage 2” (CRASH-2) trial, a large multicenter randomized, placebo-controlled trial evaluating the effects of TXA in patients with trauma, were published [6,7]. The study found that early administration of TXA (treatment within the first 3 h after injury) safely reduced the risk of death in bleeding trauma patients by a third and was highly cost-effective.

Nowadays, TXA is used both pre-hospital and in-hospital as an anti-fibrinolytic drug to treat or prevent hyperfibrinolysis in patients with severe trauma or undergoing major orthopedic surgery. In the case of severe trauma, TXA is usually administered systemically, whereas in elective orthopedic surgery, TXA may be applied systemically or locally at the site of bleeding [8,9].

Despite its frequent application, however, concerns remain about possible effects on skeletal tissue, especially during elective surgery, as bone integrity and adequate bone regeneration is of utmost importance in orthopedic trauma surgery patients [10,11,12]. Bone remodeling and regeneration following injury or surgery depends on the balanced activities of the key skeletal cell types, bone-forming osteoblasts, and bone-resorbing osteoclasts [13,14]. In addition, macrophages have also been recognized not only to significantly contribute to the regulation of bone turnover but also fracture repair [15,16]. In this regard, a direct effect of TXA on osteoblasts, osteoclasts, and macrophages is largely unknown. A potential negative or even toxic effect of systemically or locally applied TXA on bone cells could thus significantly delay regeneration and rehabilitation and may impair overall bone turnover in patients receiving TXA. While TXA was found not to affect lumbar spine fusion in mice [17], results reported in the abstract suggested that the related antifibrinolytic agent, caproic acid, inhibits osteoblast differentiation and matrix mineralization in vitro [18,19]. Together, currently available data are insufficient to allow definite conclusions regarding the effects of TXA on bone cells.

In this study, we hypothesized that TXA modulates the differentiation and function of bone cells. Thus, we employed highly standardized cell culture systems of osteoblasts, osteoclasts, and macrophages and analyzed cell differentiation, matrix mineralization, and gene expression as primary outcome measures.

## 2. Results

**Figure 1:**
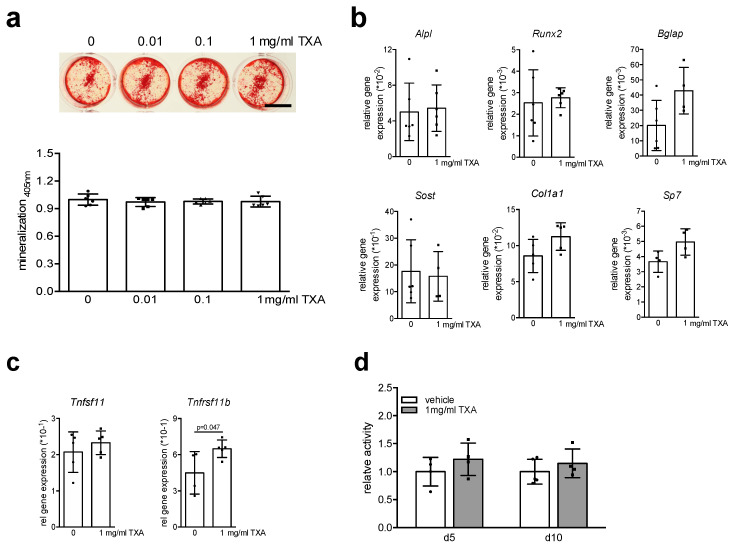
TXA does not affect the differentiation or function of calvaria-derived osteoblasts. (**a**) Representative alizarin red stainings of calvaria-derived osteoblasts from WT mice differentiated in the presence of indicated concentrations of TXA for 10 days in osteogenic medium. Scale bar 10 mm. The quantification of extracellular matrix mineralization is indicated below. (**b**) qRT-PCR expression analysis for the indicated genes in calvaria-derived osteoblasts at day 10 of osteogenic differentiation, stimulated with TXA (1 mg/mL) during the entire course of cell differentiation. (**c**) qRT-PCR expression analysis for the indicated genes in the same samples. (**d**) MTT proliferation assay of calvaria-derived osteoblasts stimulated with 1 mg/mL TXA for 6 h at the indicated days of differentiation. For (**a**–**d**), *n* = 4–6 independent cultures per group were used, as indicated by individual data points. Data presented are means ± SD. Gene abbreviations: runt-related transcription factor 2 (*Runx2*), osterix (*Sp7*), alkaline phosphatase (*Alpl*), alpha-1 type I collagen (*Col1a1*), osteocalcin (*Bglap*), sclerostin (*Sost*), Rankl (*Tnfsf11*), osteoprotegerin (*Tnfrsf11b*).

To evaluate the direct effect of TXA on osteoblasts, primary osteoblasts were enzymatically isolated from the calvaria of murine newborn pups and differentiated with osteogenic medium in the presence of 0.01, 0.1, and 1 mg/mL TXA for 10 days. As assessed by alizarin red staining of culture plates, long-term TXA treatment did not affect extracellular matrix mineralization at any of the tested concentrations (Figure 1a). To test whether potential effects of TXA on osteoblasts can be observed on the molecular level, we studied gene expression of osteoblast markers in primary osteoblast cultures differentiated with osteogenic medium and 1 mg/mL TXA for 10 days. Similar to extracellular matrix mineralization, long-term TXA treatment did not affect the expression of the key osteoblast transcription factors runt-related transcription factor 2 (*Runx2*) and osterix (*Sp7*) (Figure 1b). Likewise, the expression of the hallmark osteoblast markers alkaline phosphatase (*Alpl*), alpha-1 type I collagen (*Col1a1*), and osteocalcin (*Bglap*), as well as the Wnt-inhibitor Sclerostin (*Sost*), was not significantly affected by long-term TXA treatment. Monitoring the expression of *Tnfsf11* (encoding pro-resorptive Rankl) and *Tnfrsf11b* (encoding anti-resorptive osteoprotegerin), we observed TXA to induce *Tnfrsf11b* expression, whereas *Tnfsf11* levels remained unchanged (Figure 1c). Finally, TXA did not affect cell proliferation, as assessed by MTT viability assay (Figure 1d). Collectively, the data indicate that TXA does not directly affect calvaria-derived osteoblasts.

**Figure 2:**
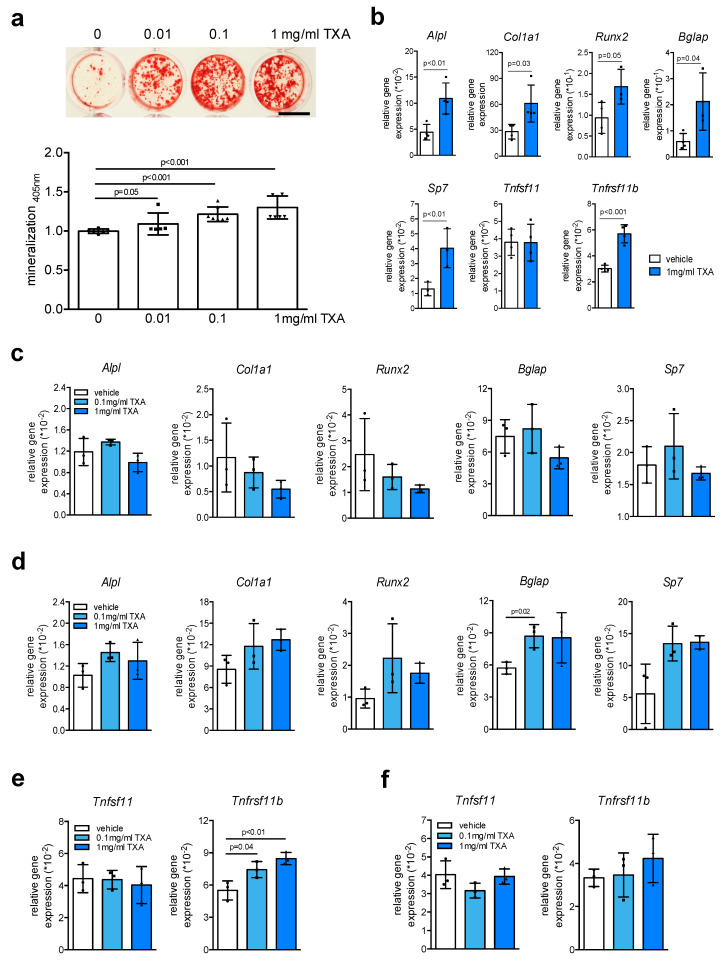
TXA promotes extracellular matrix mineralization. (**a**) Representative alizarin red stainings of bone marrow-derived osteoblasts from WT mice differentiated in the presence of indicated concentrations of TXA for 10 days in osteogenic medium. Scale bar 10 mm. The quantification of extracellular matrix mineralization is indicated below. (**b**) qRT-PCR expression analysis for the indicated genes in bone marrow-derived osteoblasts at day 10 of osteogenic differentiation, stimulated with TXA (1 mg/mL) during the entire course of cell differentiation. (**c**,**d**) qRT-PCR expression analysis for the indicated genes in bone marrow-derived osteoblasts at day 2 (**c**) and day 10 (**d**) of osteogenic differentiation, stimulated with TXA for 6 h at the indicated concentrations after serum starvation overnight. (**e**) qRT-PCR expression analysis for the indicated genes in the same cells at day 2 or (**f**) day 10 of differentiation. For (**a**–**f**), *n* = 3–4 independent cultures per group were used. Data presented are means ± SD. Gene abbreviations: runt-related transcription factor 2 (*Runx2*), osterix (*Sp7*), alkaline phosphatase (*Alpl*), alpha-1 type I collagen (*Col1a1*), osteocalcin (*Bglap*), sclerostin (*Sost*), Rankl (*Tnfsf11*), osteoprotegerin (*Tnfrsf11b*).

As in vivo bone remodeling and formation rely on the close communication of osteoblasts with a broad range of different cell types, we next tested the effects of TXA in osteoblast cultures derived from murine bone marrow, resembling the typical cell composition of fracture hematoma. In these cultures, we observed a dose-dependent effect of long-term TXA stimulation to increase extracellular matrix mineralization (Figure 2a). These results were confirmed by gene expression analysis at day 10 of osteoblast differentiation, where we found long-term TXA treatment to induce the expression of all osteoblast markers tested, including *Runx2*, *Sp7*, *Alpl*, *Col1a1*, and *Bglap* (Figure 2b). Likewise, we found long-term TXA treatment to increase the expression of *Tnfrsf11b*, whereas *Tnfsf11* mRNA was not affected. To evaluate whether TXA exerts a direct, osteogenic effect on osteoblast precursors, we next stimulated bone marrow-derived osteoblasts at an early stage of differentiation (day 2) with TXA for 6 h and monitored gene expression. In contrast to the effects of long-term stimulation, short-term treatment of these cultures with TXA did not significantly affect gene expression of osteoblast markers (Figure 2c). The same was observed when we stimulated terminally differentiated, bone marrow-derived osteoblasts at day 10 with TXA (Figure 2d), indicating that TXA enhances bone formation through other mechanisms than osteogenic induction. TXA treatment for 6 h at day 2 of differentiation resulted in increased expression of *Tnfrsf11b* (Figure 2e), which however was not detectable at day 10 of differentiation (Figure 2f). The expression of *Tnfsf11*, however, was not altered in bone marrow-derived osteoblasts.

**Figure 3:**
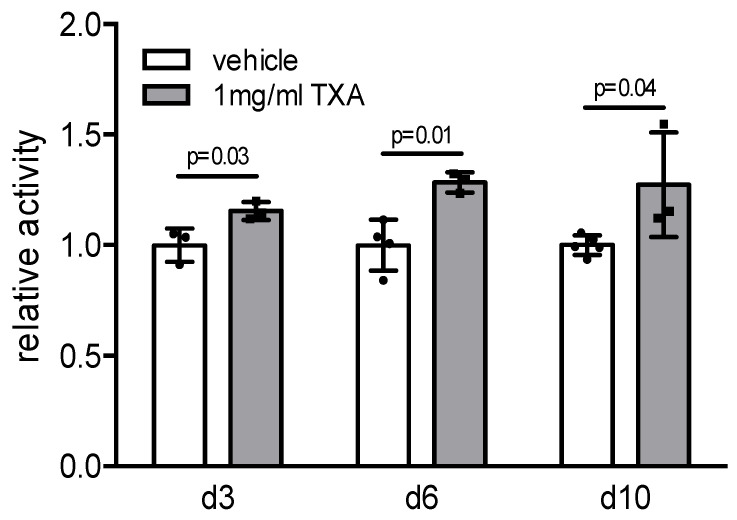
TXA enhances cell proliferation of bone marrow-derived osteoblasts. MTT proliferation assay of bone marrow-derived osteoblasts stimulated with 1 mg/mL TXA for 6 h at the indicated time points; *n* = 3–5 independent cultures per group were used. Data presented are means ± SD.

Monitoring cell metabolism, we employed MTT assays in bone marrow-derived osteoblasts stimulated with TXA for 6 h at different stages of differentiation (day 3, 6, and 10). Here we observed a significantly increased cell proliferative rate in TXA-treated osteoblasts at all time points analyzed, indicating that TXA exerts an osteoanabolic effect on bone formation through the stimulation of osteoblast proliferation (Figure 3).

**Figure 4:**
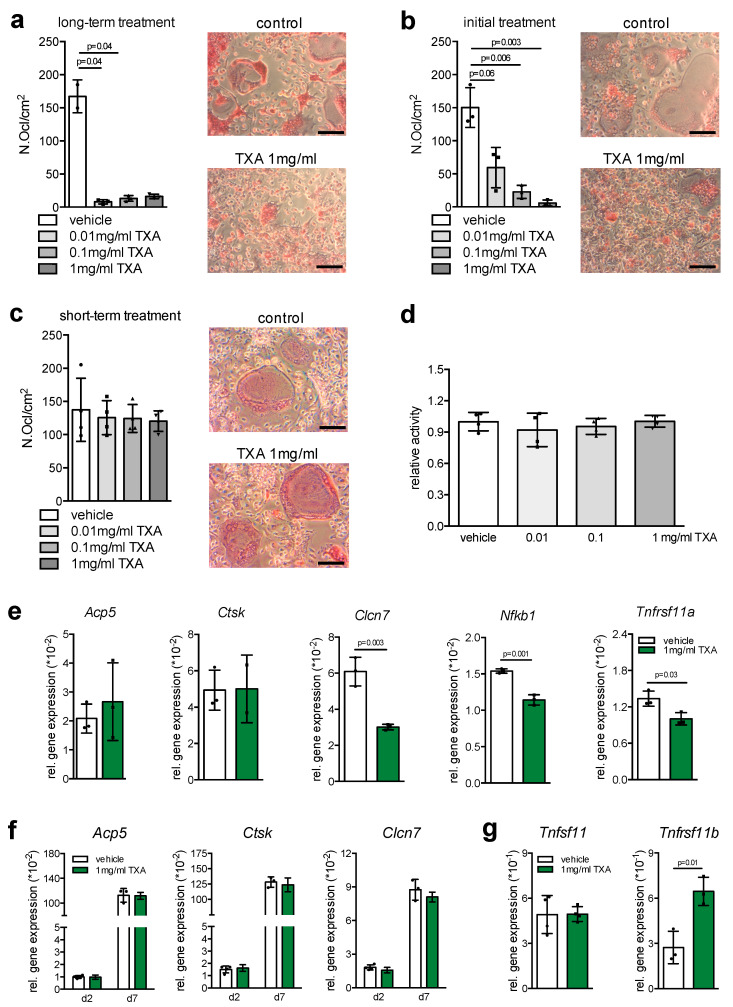
Inhibition of early osteoclastogenesis through TXA. (**a**) TRAP activity staining of WT bone marrow cells at day 7 of differentiation, cultured in the presence of M-CSF and RANKL and continuous exposure to vehicle or TXA at the indicated concentrations. Scale bars = 50 μm. The quantification of osteoclast numbers per viewing field is depicted on the left (Ocl.N./VF). (**b**) TRAP activity staining of WT bone marrow cells at day 7 of differentiation, cultured in the presence of M-CSF and RANKL, and exposure to vehicle or TXA at the indicated concentrations only at day 1 of osteoclast differentiation. The quantification of osteoclast numbers per viewing field is depicted on the left (Ocl.N./VF). (**c**) TRAP activity staining of WT osteoclasts at day 7 of differentiation, cultured in the presence of M-CSF and RANKL and exposed to vehicle or TXA at the indicated concentrations for 24 h. (**d**) MTT proliferation assay of WT osteoclasts at day 7 of differentiation stimulated with the indicated concentrations of TXA for 6 h. (**e**) qRT-PCR expression analysis for the indicated genes in bone marrow-derived osteoclasts at day 7, stimulated with TXA (1 mg/mL) during the entire course of cell differentiation. (**f**) qRT-PCR expression analysis for the indicated genes in bone marrow-derived osteoclasts at the indicated stages of cell differentiation, stimulated with TXA (1 mg/mL) for 6 h. (**g**) qRT-PCR expression analysis for the indicated genes in bone marrow-derived osteoclasts at day 5 of differentiation, stimulated with TXA (1 mg/mL) during the entire course of cell differentiation. For (**a**–**g**), *n* = 3–4 independent cultures per group were used. Data presented are means ± SD. Gene abbreviations: chloride channel 7 (*Clcn7*), nuclear factor kappa B subunit 1 (*Nfkb1*), receptor activator of nf-κb (*Tnfrsf11a*), tartrate-resistant acid phosphatase (*Acp5*), cathepsin K (*Ctsk*).

As bone resorption represents an essential mechanism to maintain bone quality through removal of damaged or aged bone tissue, we next cultured bone marrow cells undergoing osteoclast differentiation with continuous TXA exposure. Here, we detected a striking reduction in osteoclast formation at all concentrations tested (Figure 4a). This effect was also observed when bone marrow cells were treated only once with TXA at the beginning of osteoclast differentiation, indicating that the inhibitory effect of TXA on osteoclastogenesis affects early osteoclast formation (Figure 4b). In contrast, stimulation of osteoclast cultures with TXA for 24 h did not affect osteoclast numbers at day 7 of differentiation (Figure 4c). Likewise, stimulation of osteoclast cultures with TXA for 6 h caused no alteration in cell viability as assessed by MTT assay (Figure 4d).

In line with the notion that TXA inhibits early osteoclastogenesis, long-term TXA treatment resulted in a significantly decreased expression of the key osteoclast markers chloride channel 7 (*Clcn7*), nuclear factor kappa B subunit 1 (*Nfkb1*), and receptor activator of nf-κb (*Tnfrsf11a*), whereas tartrate-resistant acid phosphatase (*Acp5*) and cathepsin K (*Ctsk*) were not affected (Figure 4e). In order to understand this phenomenon on the molecular level, we next analyzed the expression of key osteoclast markers after short-term treatment with TXA (6 h) at early (day 2) and late (day 7) stages of osteoclast differentiation. Here we did not detect significant alterations in the expression of *Acp5*, *Clcn7*, or *Ctsk* upon short-term stimulation with TXA at the respective time points (Figure 4f). As this pointed towards an indirect effect of TXA on osteoclastogenesis, we monitored the expression of *Tnfsf11* (encoding pro-resorptive Rankl) and *Tnfrsf11b* (encoding anti-resorptive osteoprotegerin) in these cultures, since they also contain mesenchymal stems cells regulating osteoclast activity through cell-to-cell contact and in a paracrine fashion. Here we found TXA to significantly induce the expression of *Tnfrsf11b*, whereas the expression of *Tnfsf11* was not affected (Figure 4g), indicating that TXA controls osteoclastogenesis indirectly through inducing osteoprotegerin in cells of the osteoblast lineage.

**Figure 5:**
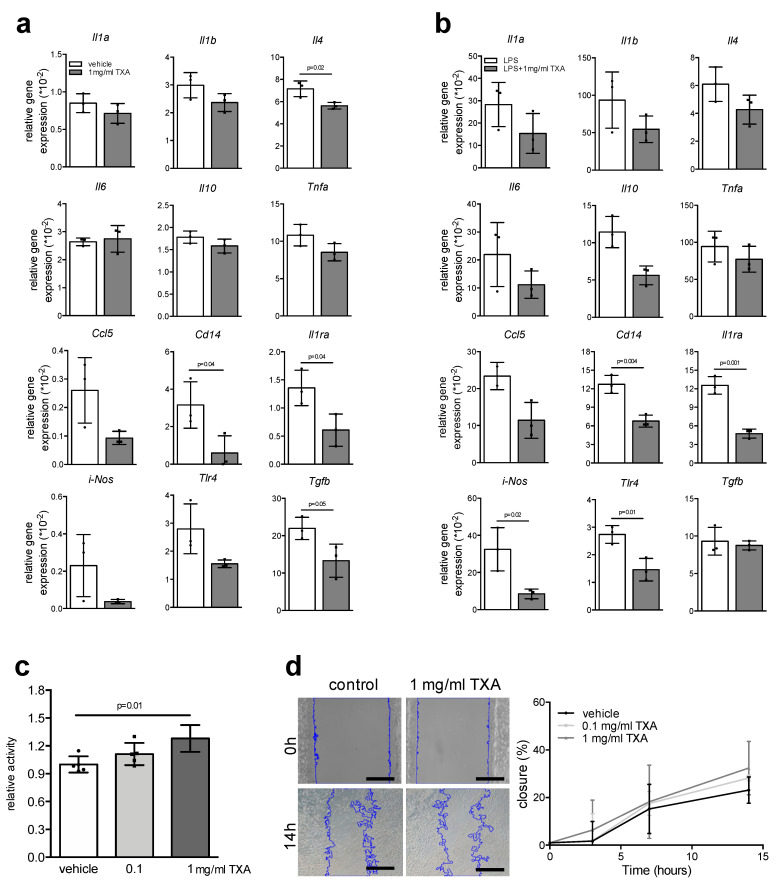
Modulation of cytokine responses through TXA in resting and activated macrophages. (**a**) qRT-PCR expression analysis for the indicated genes in bone marrow-derived macrophages at day 7 of differentiation, stimulated with TXA (1 mg/mL) for 6 h. (**b**) qRT-PCR expression analysis for the indicated genes in bone marrow-derived macrophages at day 7 of differentiation, activated with 1 μg/mL LPS and simultaneously co-stimulated with TXA (1 mg/mL) for 6 h. (**c**) MTT proliferation assay of bone marrow-derived macrophages stimulated with the indicated concentrations of TXA. (**d**) Representative images of macrophage migration assays using Ibidi chambers of the indicated groups and time points. Scale bars = 50 μm. Blue lines indicate spreading cell fronts. The quantification of cell migration is shown on the right. For (**a**–**d**), *n* = 3–5 independent macrophage cultures per group were used as indicated with individual data points. Data presented are means ± SD. Gene abbreviations: interleukin-1 alpha (*Il1a*), interleukin-1 beta (*Il1b*), interleukin-4 (*Il4*), interleukin-6 (*Il6*), interleukin-10 (*Il10)*, tumor necrosis factor alpha (*Tnfa*), CC-chemokine ligand 5 (*Ccl5*), cluster of differentiation 14 (*Cd14*), interleukin-1 receptor antagonist (*Il1ra*), inducible NO synthase (*iNos*), toll-like receptor 4 (*Tlr4*), transforming growth factor beta (*Tgfb*).

Similar to osteoblasts and osteoclasts, macrophages are of crucial importance in maintaining bone hemostasis, especially during bone regeneration following fracture. Therefore, we finally analyzed the effects of short-term TXA stimulation on gene expression in bone marrow-derived macrophages. Among all the inflammatory markers tested, we observed a significantly reduced expression of interleukin-4 (*Il4*), *Il10*, *Cd14*, interleukin 1 receptor antagonist (*Il1ra*), and transforming growth factor beta (*Tgfb*) in macrophages stimulated with TXA for 6 h (Figure 5a). Similar effects were observed in the case of macrophages activated with lipopolysaccharide (LPS), where we observed significantly decreased expression of *Il10*, *Cd14*, *Il1ra*, inducible NO synthase (*iNos*), and toll-like receptor 4 (*Tlr4*) when co-stimulated with TXA (Figure 5b). Studying cell viability, we found TXA to cause a small but significant increase in macrophage proliferation (Figure 5c). In contrast, TXA did not significantly affect the migratory behavior of macrophages, as assessed by Ibidi chamber assay (Figure 5d).

## 3. Discussion

In orthopedic surgery, the use of systemically and topically applied TXA has been expanding over the past years, with mounting evidence for relevant reductions in posttraumatic and perioperative blood loss and transfusion requirements [2,20]. However, little evidence exists regarding direct effects of TXA on bone cells, including osteoblasts, osteoclasts, and macrophages. Our study reveals that TXA exerts an osteoanabolic effect on bone marrow-derived osteoblasts, while it inhibits osteoclastogenesis and cytokine responses in macrophages.

Using pure osteoblast cultures derived from calvarial digestions, we did not detect an influence of TXA on the differentiation or function of osteoblasts. Thus, it is indeed surprising that a robust osteoanabolic effect was observed in osteoblasts derived from bone marrow flushes. This finding indicates that TXA does not directly affect osteoblasts or their precursors but mediates their effects through other cell types found within the bone marrow. Although we cannot provide direct experimental evidence, it could be possible that TXA modulates cell-to-cell communications between osteoblasts and other bone marrow cells present in these cultures [21]. Among others, these include macrophages and osteoclast precursors, which have been shown to be of crucial importance in regulating osteoblast differentiation and function through the alteration of membrane-bound and soluble factors [22,23]. Despite this open question, as a possible explanation for the increased extracellular matrix mineralization, we found TXA to stimulate cell proliferation in these cultures, whereas the expression of key osteoblastic marker genes was not affected by short-term treatment. The increased rate of cell proliferation resulted in increased matrix mineralization and enhanced expression of key osteoblast markers following long-term TXA treatment. This is in contrast to previous studies reported in the abstract, which showed variable effects on osteoblasts due to protease inhibition or an inhibitory effect of TXA on osteoblast differentiation in vitro [19,24]. However, our results are in line with in vivo studies demonstrating enhanced bone formation and decreased bone resorption in mice lacking tissue-type plasminogen activator or urokinase-type plasminogen activator, which represent the molecular target of TXA [25,26].

Investigating the effects of TXA on bone resorbing osteoclasts, we found long-term TXA treatment to potently inhibit osteoclastogenesis. This effect was also observable when osteoclast cultures were treated only once with TXA at the beginning of cell differentiation, but not when treated at late differentiation stages. Again, a direct, short-term effect of TXA on osteoclasts was not detectable, as TXA did not affect the expression of key osteoclast marker gene required for osteoclast differentiation or function. As a potential explanation, we found TXA to induce the expression of osteoprotegerin, a decoy receptor neutralizing the pro-resorptive effects of Rankl [27]. Since mesenchymal stem cells, including cells of the osteoblast lineage, are not only present in calvaria and bone marrow-derived osteoblast cultures, but also in bone marrow-derived osteoclast cultures [22], this observation provides a plausible explanation for the inhibitory effect of TXA on osteoclastogenesis.

As increasing evidence has demonstrated an essential role of macrophages in skeletal health and disease [15,16,28], we also investigated the effects of TXA on bone marrow-derived macrophages. While only minor alterations in cell proliferation and no changes in cell migration were observed, we found TXA to reduce pro- and anti-inflammatory cytokine expression in native and activated macrophages. While experimental and clinical data on potential immunomodulatory effects of TXA are limited, a previous study found TXA to lower inflammatory responses in patients with cardiopulmonary bypass surgery [29]. Likewise, TXA was reported to reduce leukocyte recruitment towards implanted biomaterials in mice [30]. Together with our findings, these results indicate a novel immunomodulatory effect of TXA on innate immune cells, whose contribution to bone remodeling and regeneration requires further mechanistic study.

The question whether the results from these in vitro experiments can be transferred to the clinic is very difficult to answer [31]. As bioavailability and effective concentration at the site of action in skeletal tissue are dependent on a broad variety of different factors, which cannot be modelled in our assays, dose–response studies are required to test required dosages for potential local and systemic applications.

Collectively, our data provide evidence for differential effects of TXA on bone cells. These include enhanced proliferation and increased extracellular matrix mineralization of bone marrow-derived osteoblasts, an inhibition of early osteoclastogenesis, and immunomodulatory effects on cytokine expression in macrophages. Thus, further pre-clinical and clinical studies are warranted to test novel treatment indications for TXA, including critical-sized bone defects, impaired bone regeneration, or fracture patients with osteoporosis.

## 4. Materials and Methods

### 4.1. Primary Bone Cells

For all experiments, female and male mice with a pure C57Bl/6J genetic background were used. All experimental protocols including cell isolation from euthanized mice were approved by the local legal representative animal rights protection authorities (Landesamt für Gesundheit und Soziales (LaGeSo) Berlin (24 Apr 2018); registered under T0131_18). Furthermore, all experiments were performed adherent to the policies and principles established by the animal Welfare Act (Federal Law Gazette I, p.1094). No experiments were performed on living mice. Primary osteoblasts were isolated by sequential collagenase and dispase digestion from calvariae of 3–5 day-old mice, cultivated in αMEM (Merck KGaA, Darmstadt, Germany) supplemented with 10% FCS, and differentiated in the presence of 25 μg/mL ascorbic acid and 5 mM β-glycerophosphate. Bone marrow-derived osteoblasts were obtained by flushing out bone marrow from 12–16 week-old female WT mice. Osteoblast differentiation was induced through stimulation with 25 μg/mL ascorbic acid and 5 mM β-glycerophosphate for 10 days. For alizarin red staining of calvaria- and bone marrow-derived osteoblasts, cells were incubated with 40 mM alizarin red staining solution (pH 4.2) for 10 min at room temperature after fixation in 90% ethanol. To quantify alizarin red incorporation, cells were washed with PBS and fixed in 90% ethanol for 1 h. After washing, cells were stained with alizarin red S solution (40 mM, pH 4.2) for 10 min. Following additional washing, the cell-bound alizarin red was dissolved in 10% acetic acid. After incubation for 30 min at room temperature and 10 min at 85 °C, the supernatant of a subsequent centrifugation step was neutralized with 10% ammonium hydroxide solution, and the absorbance was measured at 405 nm. For osteoclastogenesis, the bone marrow was flushed out of the femora from 12–16 week-old mice. Cells were plated at a density of 5 × 10^6^ cells/mL in the presence of 10 nM 1,25 dihydroxyvitamin-D3 in αMEM including 10% FCS to stimulate endogenous RANKL expression [32]. On the following day, the adherent cells were cultured for 3 days, before M-CSF (Peprotech, Hamburg, Germany) and sRANKL (Peprotech, Hamburg, Germany) were added to a final concentration of 20 and 40 ng/mL, respectively. For TRAP (tartrate-resistant acid phosphatase) activity staining, the cells were washed with PBS and then fixed for 5 min in cold methanol. After two washing steps with distilled water, the fixed cells were first dried for 2 min, before they were subjected to the TRAP-specific substrate naphthol AS-MX phosphate (Merck KGaA, Darmstadt, Germany. Primary macrophages were generated from bone marrow cells cultured in αMEM supplemented with 10% FCS and 40 ng/mL M-CSF for 7 days. Osteoblasts, osteoclast, and macrophage cultures were stimulated with TXA (Carinopharm GmbH, Elze, Germany) at the indicated concentrations (ranging from 0.01 to 1 mg/mL) and for the indicated durations. For the applied concentrations of TXA in the experiments, the TXA stock solution (100 mg/mL) was diluted as required in culture medium. Then 10 μL TXA of this stimulatory medium was added to 990 μL culture medium for stimulating or culturing bone cells with 0.01, 0.1, or 1 mg/mL TXA after washing of culture wells with PBS. For controls, an equivalent volume of vehicle was added to the culture medium. The dosages were chosen according to previous in vitro reports, which observed potent anti-fibrinolytic effects, but no cell toxicity [12,33].

### 4.2. Expression Analysis

RNA was isolated using a NucleoSpin RNA/protein kit (Macherey-Nagel, Düren, Germany), and DNase digestion was performed according to manufacturer’s instructions. Concentration and quality of RNA were measured using aNanoDrop ND-1000 system (NanoDrop Technology, Wilmington, NC, USA). Expression analysis by qRT-PCR was performed using a StepOnePlus system and predesigned TaqMan gene expression assays (Thermo Fisher Scientific, Schwerte, Germany). *Gapdh* expression was used as an internal control.

### 4.3. MTT Test

An MTT assay was used to measure cellular metabolic activity in the presence of TXA. The assay is used as an indicator of cell viability and proliferation. The colorimetric assay is based on the reduction of the tetrazolium salt (3-(4,5-dimethylthiazol-2-yl)-2,5-diphenyltetrazolium bromide or MTT) to formazan crystals by metabolically active cells. The differentiated cells were treated with 1 mg/mL MTT and incubated for 6 h. Supernatants were removed, and isopropanol was added to each well. The absorbance of formazan was read at 570 nm with a 650 nm reference filter using an ELISA micro plate reader.

### 4.4. Migration Assay

The migration assay was performed in a Culture-Insert 2 Well µ-Dish 35 mm (Ibidi chambers, Gräfelfing, Germany) according to the manufacturer’s instructions. Macrophages were seeded at a density of 5 × 10^6^ cells/mL with αMEM supplemented with 10% FCS. The cells were differentiated for the indicated duration. The growth medium was replaced with stimulating medium containing TXA, and cells were further incubated for 14 h. Macrophages were photographed at defined time points during the course of the experiment, and the gap was measured using NIH ImageJ software.

### 4.5. Statistical Analysis

Data were analyzed by GraphPad Prism 8. Two-group comparisons were analyzed by unpaired Student’s *t* tests. Bonferroni adjustment was employed in cases of multiple comparisons; *p* < 0.05 was considered statistically significant.

## 5. Conclusions

Overall, our data provide in vitro evidence of the differential effects of TXA on bone cells. Hereby, the anti-fibrinolytic drug showed osteoanabolic, anti-resorptive, and anti-inflammatory effects on osteoblasts, osteoclasts, and macrophages, respectively, suggesting novel treatment applications.

## Data Availability

All relevant data is presented in the manuscript, raw data is available upon request from the corresponding author.

## Abbreviations

Acp5	tartrate-resistant acid phosphatase
Alpl	alkaline phosphatase
Bglap	osteocalcin
Clcn7	chloride channel 7
Col1a1	alpha-1 type I collagen
CRASH-2	clinical randomization of an antifibrinolytic in significant hemorrhage 2
Ctsk	cathepsin K
Il1ra	interleukin 1 receptor antagonist
iNos	NO synthase
LaGeSo	Landesamt für Gesundheit und Soziales
LPS	lipopolysaccharide
Nfkb1	nuclear factor kappa B subunit 1
Runx2	runt-related transcription factor 2
Sost	sclerostin
Sp7	osterix
Tgfb	transforming growth factor beta
Tlr4	toll-like receptor 4
Tnfrsf11a	receptor activator of nf-κb
TXA	tranexamic acid
